# Curcumin and tumor immune-editing: resurrecting the immune system

**DOI:** 10.1186/s13008-015-0012-z

**Published:** 2015-10-12

**Authors:** Sayantan Bose, Abir Kumar Panda, Shravanti Mukherjee, Gaurisankar Sa

**Affiliations:** Division of Molecular Medicine, Bose Institute, P-1/12, CIT Scheme VII M, Kolkata, 700054 India

**Keywords:** 3-Es, Curcumin, Hallmarks of cancer, Nanocurcumin, Tumor immune-editing, Tumor immunesurveillance

## Abstract

Curcumin has long been known to posses medicinal properties and recent scientific studies have shown its efficacy in treating cancer. Curcumin is now considered to be a promising anti-cancer agent and studies continue on its molecular mechanism of action. Curcumin has been shown to act in a multi-faceted manner by targeting the classical hallmarks of cancer like sustained proliferation, evasion of apoptosis, sustained angiogenesis, insensitivity to growth inhibitors, tissue invasion and metastasis etc. However, one of the emerging hallmarks of cancer is the avoidance of immune system by tumors. Growing tumors adopt several strategies to escape immune surveillance and successfully develop in the body. In this review we highlight the recent studies that show that curcumin also targets this process and helps restore the immune activity against cancer. Curcumin mediates several processes like restoration of CD4^+^/CD8^+^ T cell populations, reversal of type-2 cytokine bias, reduction of Treg cell population and suppression of T cell apoptosis; all these help to resurrect tumor immune surveillance that leads to tumor regression. Thus interaction of curcumin with the immune system is also an important feature of its multi-faceted modes of action against cancer. Finally, we also point out the drawbacks of and difficulties in curcumin administration and indicate the use of nano-formulations of curcumin for better therapeutic efficacy.

## Background

Turmeric is one of the most widely used spice ingredient, derived from *Curcuma longa*, of the *Zingiberacea* (Ginger) plant family. Some fractions of turmeric, collectively known as curcuminoids (curcumin, demethoxycurcumin and bisdemethoxycurcumin) are considered to be the active compounds. Curcumin or diferuloylmethane, having molecular weight 368.38, is primary active polyphenolic compounds studied in a host of areas. It is an orange-yellow, crystalline powder and insoluble in water; however, it is highly soluble in ethanol and DMSO [1]. It is used as a spice to give the specific flavor and yellow color to curry. Curcumin has been used extensively in Ayurvedic medicine for centuries in India and South Asia, as it is nontoxic and has several beneficial properties like anti-oxidant, analgesic, anti-inflammatory and antiseptic activity. Curcumin has been used as a traditional medicine to treat a spectrum of diseases like rheumatism, body ache, skin diseases, intestinal worms, diarrhea, intermittent fevers, hepatic disorders, biliousness, inflammations, constipation, leukoderma, amenorrhea, arthritis, colitis and hepatitis [2–5]. More recently curcumin has been found to have anti-cancer properties that affect a variety of biological pathways involved in mutagenesis, oncogene expression, cell cycle regulation, apoptosis, angiogenesis and metastasis [3–5]. Several studies were conducted to explore the anti-cancer properties of curcumin and it was shown that curcumin modulates multiple cell signaling pathways which include cell proliferation (Cyclin D1, c-MYC), cell survival (BCL-2, BCL-XL, FLIP, XIAP, C-IAP1), apoptosis or cell death (Caspase-8, 3, 9), as well as controls tumor suppressor pathway (p53, p21) death receptor pathway (DR4, DR5), mitochondrial pathways, and protein kinase pathway (MAPK, JNK, AKT, and AMPK), thereby affecting tumor cell growth [4, 6–8].

## Curcumin against the hallmarks of cancer

Recently it was suggested that tumors share several common traits (hallmarks) during malignancy that govern the transformation of normal cells to cancer cells. In 2000 Hanahan and Weinberg first proposed that six biological properties of cancer cells comprise the hallmarks of cancer that are required for the multistep development of human cancer. Interestingly, curcumin can inhibit all the six major capabilities of cancer cells and restricts tumor outgrowth in the host [9].

### Curcumin perturbs proliferation signalling

Curcumin inhibits several cell proliferation signalling pathways that are relentlessly upregulated in the progression of cancer. Curcumin inhibits the expression of nuclear factor NFκB that regulates cell proliferation, metastasis, angiogenesis, apoptosis and resistance to chemotherapy [10]. Curcumin-induced down-regulation of NFκB is mediated through suppression of IκB kinase activation. The proliferation signaling cascades such as PI3K, AKT, mTOR, AP1 (JUN and FOS), JNK, JAK-STAT, PKC, CMYC, MAPK, ELK, CDKs, iNOS and Wnt/β-catenin which are also suppressed by curcumin further confirmed that it is one of the crucial molecule that prevents cancer progression by targeting multiple cell proliferation signalling. Curcumin also down-regulates the expression of Cyclin D1, the proto-oncogenes that are overexpressed in several types of cancer and plays a crucial role in cell cycle progression and proliferation [11, 12].

### Curcumin causes growth suppression

In addition to capabilities of inducing and sustaining positive growth stimulatory signals, cancer cells must also avoid the mechanisms that negatively regulate cell proliferation by predominantly inhibiting the function of tumor suppressor genes. TP53 is the most crucial protein that operates on central regulatory circuits which govern the decision of cells whether to proliferate or undergo active senescence and trigger apoptosis program. Several in vitro and in vivo studies confirmed that curcumin upregulates the expression of TP53 and induces apoptosis [13]. Curcumin also inhibits phosphorylation of RB (Retinoblastoma), another important tumor suppressor protein that also plays an important role in cell cycle process [14]. Curcumin inhibits EGF- and EGFR-mediated signalling pathway that is overexpressed in breast tumor and is involved in cancer progression [15, 16]. Curcumin also blocks excessive TGFβ receptor signalling that induces epithelial to mesenchymal transition during invasion and metastasis process [17, 18].

### Curcumin in recovering the resistance towards cell death

Tumor cells exploit a variety of strategies to limit or circumvent apoptosis. During tumor progression, the tumor suppressor protein, TP53 is depleted thus hampering its critical function as damage sensor and activator of apoptosis-inducing circuitry. Alternatively, tumors may achieve similar ends by increasing expression of anti-apoptotic regulators (BCL-2, BCL-XL) or survival signals (IGF1/2), or down regulating pro-apoptotic factors (BAX, BIM, PUMA), or by short-circuiting the extrinsic ligand-induced death pathway [19]. Curcumin elicits both TP53-dependent and -independent cancer cell apoptosis. The pro-apoptotic molecules such as BAX, BIM, PUMA are upregulated whereas anti-apoptotic partners like BCL2, BCL-XL, Survivin are down-regulated by curcumin that simultaneously activates Caspases and induces apoptosis or programmed cell death [20–23]. Curcumin also activates lysosomal proteases, phosphatases and lipases that trigger autophagy-mediated cell death [24, 25].

### Curcumin prevents angiogenesis

Like a normal cell, tumor also requires nutrients as well as oxygen and releases excess amounts of carbon dioxide for maintaining uncontrolled outgrowth. The tumor-generated angiogenesis process, fulfil all these essential needs. The angiogenic factors like VEGF and angiopoietin induces and operate overall neo-angiogenesis process. Curcumin constrains VEGF and angiopoietin overexpression and prevents angiogenesis process by cutting off food and oxygen supply to the cancer cells [26]. Curcumin also inhibits VEGF receptor (VEGFR1 and VEGFR2) expression, thereby blocking VEGF/VEGFR-mediated signalling pathway to restrict angiogenesis [13].

### Curcumin restricts replicative immortalities

The maintenance of telomere region is another essential hallmark that is required for relentless cell growth and cell senescence. The telomerase is activated during cancer progression which prevents telomere shortening and activate cell proliferative signal continuously. Curcumin inhibits human telomerase (hTERT) activities and down-regulates *hTERT*-mRNA expression leading to telomere shortening. Therefore curcumin targets telomerase activities and controls replicative cell senescence and mortality that ultimately regulate uncontrolled cell proliferative potential of cancer [27].

### Curcumin constrains activation of metastasis and invasion

**Fig. 1:**
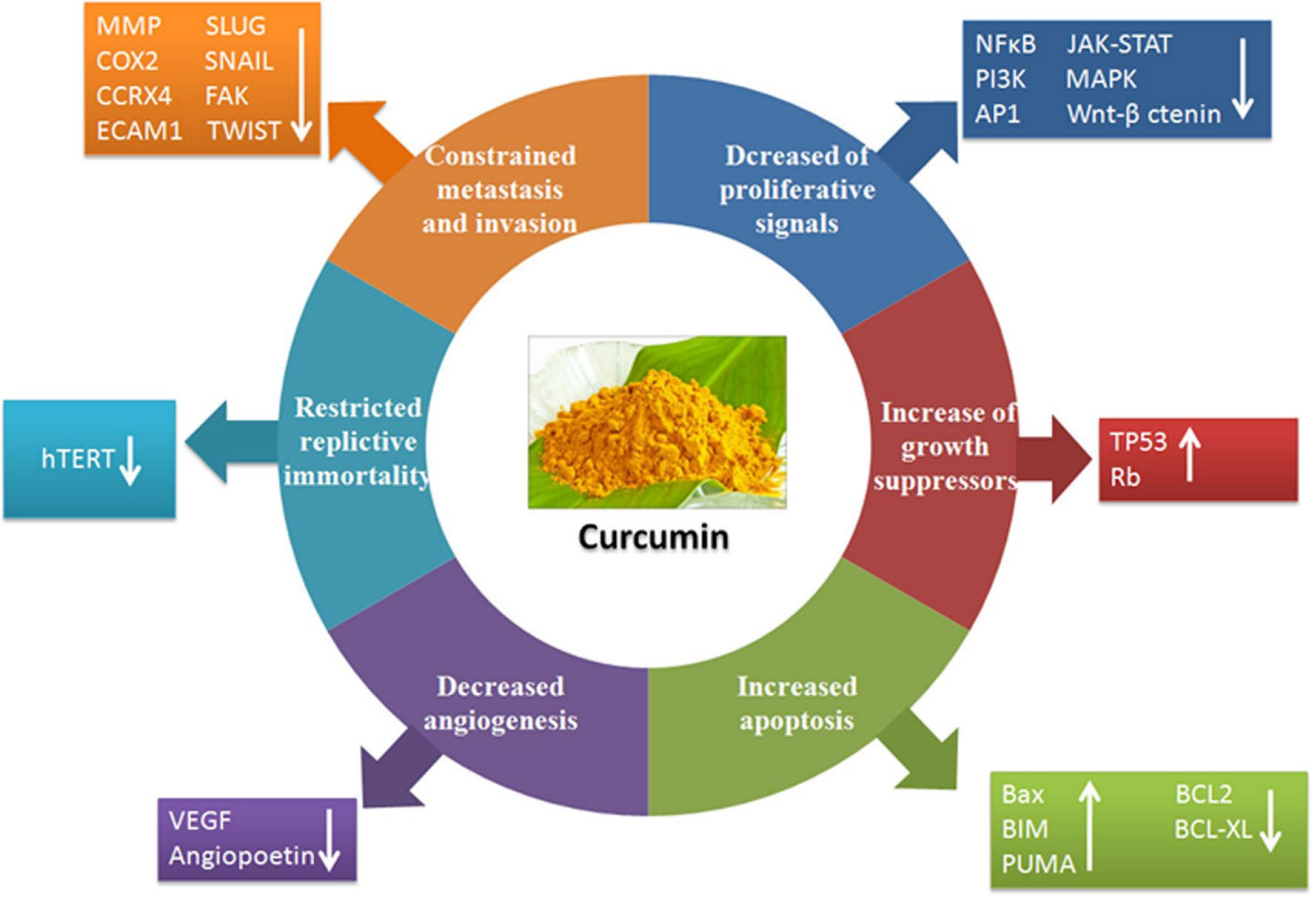
Curcumin targets the classical hallmarks of cancer: curcumin has been shown to target all the classical hallmarks of cancer. It reduces proliferative signals by interfering with pathways like NFκB, PI3K, MAPK etc. It also restores the levels of growth suppressors like TP53 and retinoblastoma protein (RB). Curcumin increases pro-apoptotic proteins like BAX, BIM, PUMA while decreasing anti-apoptotic proteins like BCL-2, BCL-XL, thus promoting apoptosis of cancer cells. Curcumin reduces angiogenesis by decreasing VEGF and angiopoetin and interfering with VEGFR signaling. Curcumin also restricts replicative immortality by reducing activity of human telomerase (hTERT). Finally curcumin reduces metastasis by targeting a host of invasion and cell adhesion related molecules like MMP, CXCR4, SLUG, SNAIL etc.

Tumor continues its invasive outgrowth and migrates to other distant sites by invading extracellular matrix via metastasis and invasion. Curcumin significantly inhibits cell migration, invasion, and colony formation in vitro and reduces tumor growth and metastasis in vivo. Curcumin down-regulates several invasion, cell adhesion and extracellular matrix molecules such as matrix metalloprotease, CCRX4, COX2, ELAM1, ECAM1 that are essential for sustaining metastasis [28]. In addition, several reports also suggested that curcumin hinders the activities of SLUG, SNAIL, FAK, TWIST and other essential transcription factors that play a crucial role in metastasis process [29]. Recently, it was found that curcumin inhibits breast cancer stem cell migration by amplifying E-cadherin/beta-catenin negative feedback loop [30] (Fig. 1).

## Avoidance of immune system: an emerging hallmark of cancer

In order to restrict potential tumor outgrowth the vertebrates possess distinct and special class of cells that can recognize and elicit specific immune response to eradicate neoplastic cells from the host body. The tumor cells are smart enough and exploit several strategies to escape immune surveillance and thwart the immune system to grow continuously and establish tumor immune evasion. The tumor-associated antigens (TAA) are not specifically neo-antigens that are exclusively expressed in tumor cells; rather they are tissue differentiation antigens also expressed in certain normal healthy cells. The nonspecific tumor antigens do not elicit proper immune responses and they are also concealed within the stroma. The innate immunity which mainly consists of antigen presenting cells (dendritic cells, macrophages) and natural killer cells (NK and NKT cells) become tolerogenic and are depleted due to apoptosis at the advanced stages of cancer. The adaptive immune response which mainly comprises of T cells (CTLs and Th1 cells), undergo apoptosis and the presence of immunosuppressive cytokines renders them unresponsive to interactions with antigen presenting cells [31, 32]. This creates an environment that is suitable for tumor outgrowth [33, 34]. In addition, release of several immunosuppressive factors induces generation of T-regulatory cell, tolerogenic macrophages and dendritic cells that accelerate the tumor immune evasion process rapidly. The immune-surveillance strategy becomes paralyzed and subsequently helps in the unrestricted growth of tumor cells [35].

In the last decades, research has also progressed about using curcumin not only as a therapeutic agent that targets several signalling-pathways in cancer but also as an immune modulator that boosts the immune system so that destruction and elimination of cancer cells from the host occurs at an early stage thereby preventing its disastrous outgrowth. In this review, we will discuss the immune editing process that is involved in tumor immune evasion and the role of curcumin to re-establish tumor immune surveillance from tumor immune escape.

### The 3E’s of immunoediting

It has been an age-old hypothesis that the immune system can recognize the formation of nascent tumors in the body and combat against them. Experimental evidences have poured in through the years to strengthen this hypothesis and the process has been referred as cancer immune surveillance. Finally, the necessity of avoiding the immune destruction for cancers to develop in the body was recognized as a hallmark of cancer development by cancer biologists Hannahan and Weinberg in [36]. The first prediction about cancer immune-surveillance was put forward by Paul Ehrlich as early as in 1909. Ehrlich hypothesized that the immune system must be preventing the growth of tumors, which would otherwise be occurring at a much higher frequency [37]. Further arguments were put forward by Burnet and Thomas about the immunesurveillance hypothesis in the 1950s [38, 39]. However the immunesurveillance process was difficult to establish experimentally, because it was an essentially invisible process, naturally occurring in the body without profound manifestations. Hence the debate regarding the existence of such mechanisms continued for a few more decades [40]. The development of sophisticated experimental techniques, especially knock-out mice with specific immunodeficiencies finally provided a stronger ground for theories regarding cancer immunesurveillance. In the 1990s, series of experiments involving tumor development in mice, deficient in particular components of immune system started providing a clearer picture of the molecular nature of immunesurveillance and its role in preventing tumor development [41, 42]. However, growing evidence suggests that the interaction between immune system and cancer is a more dynamic process and immunesurveillance is only a part of it. Interactions between immune system and tumor cells may also lead to development of a population of low immunogenic cells, that are capable of escaping from the immunesurveillance and develop into detectable tumors [43]. These observations lead to the formulation of a broader model termed as immunoediting, put forward by Dunn et al. [44]. The cancer immunoediting model not only incorporates immune surveillance but also the dynamic interactions of tumor with both adaptive and innate branches of immune system that edit and sculpt the intra-tumoral landscape. The immunoediting model serves as the most fundamental and comprehensive explanation of the importance of immune system in the war against cancer. A detailed understanding of these mechanisms is necessary for designing effective immunotherapies against cancer. The immunoediting process has mainly been divided into three phases: Elimination, Equilibrium and Escape; which are together referred as the three E’s of immunoediting. Each process represents a dynamic state of interaction between the immune system and tumor cells that may lead to either development or prevention of cancer. The three states are briefly discussed below:

*Elimination* The immune system carries out a constant surveillance process by which immune cells recognize and try to eliminate nascent tumors in the body [45]. During the early stages of tumorigenesis, transformed oncogenic cells display tumor-specific signals and antigens that are recognized by the immune system [46]. Both innate and adaptive immune systems are involved in the elimination process. During the growth of tumor, it requires blood supply, hence causing remodeling of surrounding stromal cells and formation of new blood vessels. This results in release of inflammatory cytokines like IFNγ and IL12 from tumor cells, surrounding stromal cells and macrophages. These attract cells of the innate immune system like the NK, NKT and γδ T cells leading to perforin-, FASL- and TRAIL-mediated killing of tumor cells [47, 48]. The pro-inflammatory conditions also promote maturation of dendritic cells which ingest tumor-associated antigens and present them to the adaptive immune system. The presented antigens activate the CD4^+^ T cell which in turn recruit TAA-specific CD8^+^ T cells that lead to further killing of tumor cells [49]. In the elimination phase, the reactive immune cells successfully eradicate nascent tumors and protect the host body. Hence in this case the war is won by the immune system as it successfully blocks tumor formation.

*Equilibrium* Some tumor cells may be resistant enough to withstand the attack by immune cells and enter into a stage of dormancy [50]. Tumor cells adopt variety of mechanisms to thwart the constant assault by immune cells and thereby a quiescent state is achieved where equilibrium exists between tumor proliferation and apoptosis [51]. During this phase, the constant onslaughts by the immune system may lead to selection of tumor cells that are less immunogenic. It is hypothesized that the immune system, at this stage provides a selection pressure, especially through IFNγ-mediated cytotoxicity, that kills the highly-immunogenic tumor cells but may leave a population of low-immunogenic cells that are more resistant to immune cell-mediated killing. The cancer cells are highly plastic, accumulating a number of genetic mutations. The immune elimination process may favor the existence of phenotypes with reduced immunogenicity [52]. The dynamic interaction with the immune system shapes the outcome of the process. Depending on the circumstances, this equilibrium may shift either towards elimination of tumor cells or towards their escape from immunesurvillance. This phase is considered to be the longest phase of immunoediting and may last for months to years [53]. A practical example of the equilibrium phase is observed in organ transplant cases. One study reported the occurrence of metastatic melanoma in kidney transplant recipients from a donor, who had been previously treated for melanoma, but was considered tumor free at the time of donation. This suggested that immunosuppressive conditions in the recipients may have facilitated the growth of tumors that were hidden or suppressed in the donor because an intact immune system in the donor kept them at an equilibrium state [54].

**Fig. 2:**
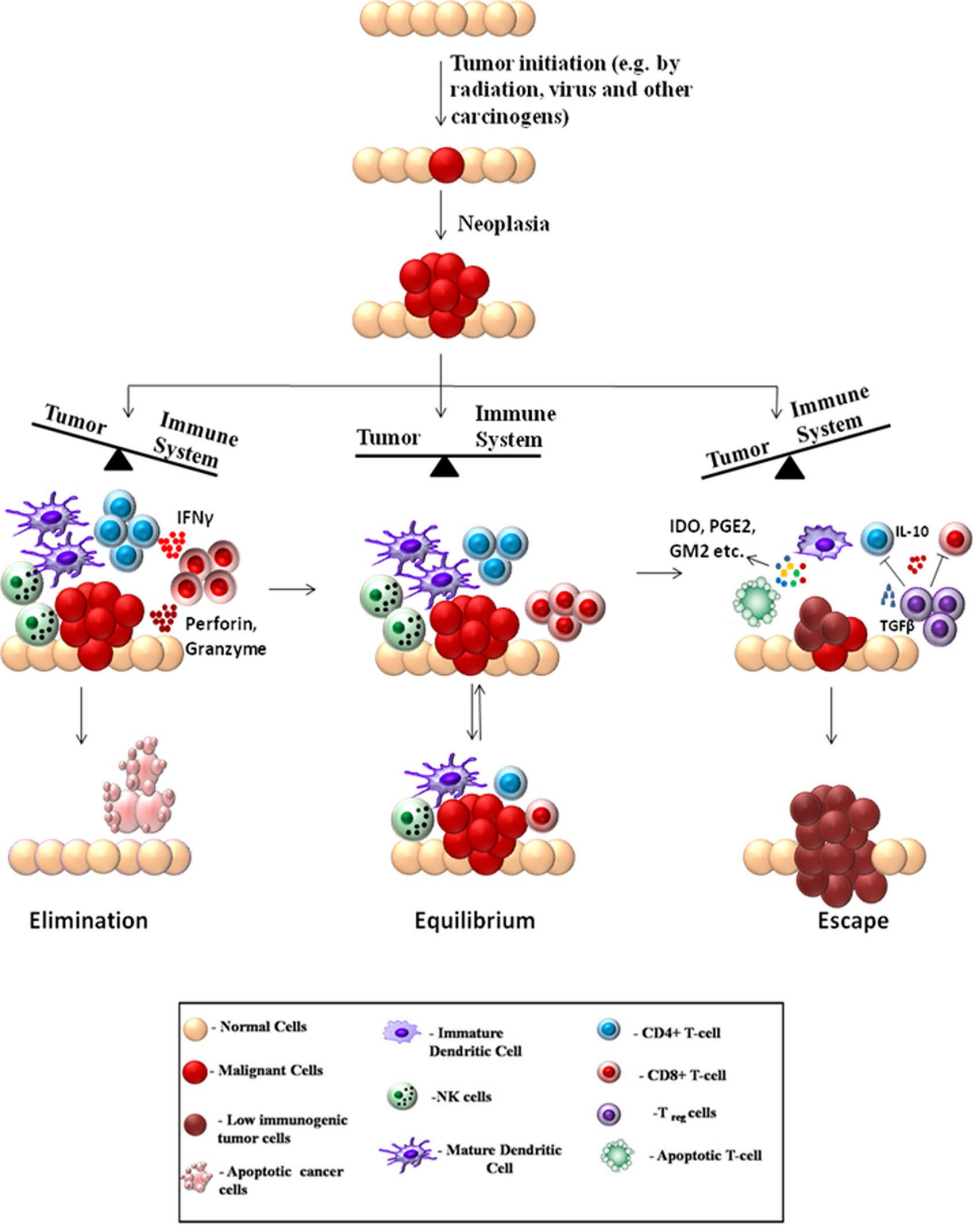
The 3 E’s of tumor immunoediting: tumor formation occurs through accumulation of mutations induced by various stress factors like radiation, virus, chemicals and other carcinogens. During initial tumor growth, the tumor cells undergo dynamic interactions with the immune system, which is called tumor immunoediting and can be divided into 3 distinct phases. I. *Elimination* In this phase the balance is tilted towards the immune system. Large numbers of CD8^+^, CD4^+^ T cells along with NK-cells, macrophages and dendritic cells mount an effective response to the tumor. Soluble factors like IFNγ, perforin, granzyme lead to tumor cell apoptosis and elimination of cancer. II. *Equilibrium* In this phase an equilibrium exists between the tumor and immune system. The immune system tries to shift the balance towards elimination whereas the tumor cells also apply mechanisms to avoid immunesurveillance. III. *Escape* The continuous assault by the immune system may lead to development of tumor cells that are less immunogenic and can avoid the immune system. The tumor has several strategies to escape the immune system; these include induction of T cell apoptosis, blocking dendritic cell maturation and promoting generation of immunosuppressive Treg cells. Hence the balance shifts towards the tumor and tumor development can occur unhindered

*Escape* The escape phase ensues when the battle is won by the tumor cells and is marked by development of clinically detectable tumors [55, 56]. The high-plasticity of tumor cells allow them to modify themselves enough to avoid the immune system. An important strategy of tumor cells to avoid destruction by immune system is to create an immunosuppressive environment by secretion of highly immunosuppressive cytokines such as TGFβ, IL10 [57]. Some tumor cells overproduce molecules like galectin, indoleamine 2-3-dioxygenase, which block T cell response and induce T cell apoptosis. They also release pro-inflammatory signals which block dendritic cell maturation [58, 59]. Another important strategy for immune escape is the induction of CD4^+^CD25^+^FOXP3^+^ T-regulatory (Treg) cells. Treg cells have the ability to suppress the immune system by adding to the pool of TGFβ and IL10, induction of T cell apoptosis by IL2 depletion, decreased co-stimulation and maturation of dendritic cells [60] (Fig. 2).

### Curcumin: general effects on the immune system

Curcumin, known for its therapeutic effects, especially in cancer, is also recognized as a potent modulator of the immune system. Curcumin has been shown to exert immunomodulatory effects on several cells and organs of the immune system [61].

*Tcells* Several studies have reported that curcumin can modulate the proliferation and activation of T cells. It has been reported that curcumin reduces the proliferation of T cells induced by compounds like concanavalin A (Con A), phytohemagglutinin (PHA), and phorbol-12-myristate-13-acetate (PMA) [62]. It has also been shown to reduce IL2 production via modulation of NFκB pathway [63]. It can both suppress and stimulate the proliferation of T cells depending on the context and dose of administration. Studies by Tomita et al. have shown that curcumin can specifically block proliferation of HTLV-1 infected T cells and primary ATL cells through cell cycle arrests by down-regulating Cyclin D1, Cdk1, and Cdc25C and induction of apoptosis by down-regulating XIAP and survivin [64, 65]. Another study by Hussain et al. carried out in T cell acute lymphoblastic leukemia showed that curcumin suppresses constitutively activated targets of PI3-kinase (AKT, FOXO and GSK3) in T cells leading to the inhibition of proliferation and induction of caspase-dependent apoptosis [66]. However other study suggested that the effect of curcumin on T cells was dose-dependent; low-dose curcumin increased the proliferation of splenic lymphocytes, whereas high-dose curcumin depressed it in mice [67].

*B*-*cells* Curcumin has also been shown to regulate other cells of the immune system. It has been shown to prohibit proliferation of B-cell lymphoma cells via down-regulation of c-MYC, BCL-XL and NFκB activities [68]. It has also been reported to block Epstein Barr Virus (EBV)-induced immortalization of B-cells [69].

*Macrophages* Curcumin has been shown to modulate macrophage activities and inhibit generation of ROS in macrophages. It promotes enhanced phagocytosis of peritoneal macrophages in mice [70].

*NK cells* Curcumin is also effective against natural killer T cell lymphoma cell lines, where it promotes apoptosis by regulating the NFκB pathway and blockage of BCL-XL, Cyclin D1 etc. [71].

*Dendritic cells* Kim et al. reported that curcumin can suppress expression of CD80, CD86 and class-II antigens by dendritic cells. Curcumin also blocked the release of inflammatory cytokines like IL1β, IL6 and TNFα from LPS-stimulated dendritic cells. Curcumin was shown to modulate phosphorylation of MAPK and nuclear translocation of NFκB in dendritc cells [72].

### Curcumin and anti-tumor immune response:

Apart from the direct effect of curcumin in reducing proliferation of various immune cell or lymphomas, there are plenty of evidences suggest that curcumin can enhance anti-tumor immunity, thereby tilting the balance in favor of immune system-mediated eradication of tumor. Hence it would be interesting to envisage the role of curcumin with respect to the immunoediting process described earlier. As mentioned earlier, tumor growth is associated with escape of immunosurveillance processes and causes a general immunosuppression in the body. This is manifested by lower percentages of effector T cells (CD4^+^ and CD8^+^) and a shift from Th1 to Th2 type cytokine production, leading to decreased activity of cytotoxic T lymphocytes (CTLs) [73]. This is accompanied by an increase in levels of Treg cells which have an inhibitory effect on the immune system by secreting anti-inflammatory cytokines like TGFβ and IL10 [74]. Th1 type immune response is considered to be appropriate for fighting against cancer. IL2 and IFNγ are two Th1 type cytokines that promote survival, activation and proliferation of CTLs as well as helper T cells [75]. Hence presence of these cytokines is essential for development of robust anti-tumor responses. Th2 response on the other hand is inappropriate towards tumor as it fails to destroy tumor cells and inhibits cell-mediated immunity [76].

### Restoration of CD4^+^ and CD8^+^ T cell populations

Sa and co-workers showed that curcumin is effective in restoring populations of CD4^+^ and CD8^+^ cells in the tumor microenvironment and thereby driving the Th2 cytokine bias towards a Th1 type response again [77, 78]. Curcumin efficiently restored CD4^+^ and CD8^+^ populations in all immune compartments of tumor-bearing mice. The study also showed that curcumin administration prevented depletion of central memory and effector memory T cell. The presence of increased population of tumor infiltrating lymphocytes leads to increased tumor-cell killing, thereby eliminating the tumor from the body.

### Increased Th1 type response

The observed reduction of Th1 cytokines like IFNγ and increased type-2 cytokines like IL4 during cancer progression was also reversed by curcumin. Some reports however suggest that curcumin favors a Th2-type response while others report that curcumin promotes cancer regression by restoring Th1 immune responses [79]. Gertsch et al. for example showed that curcumin has the ability to upregulate *IFNγ* mRNA expression, which is a type-1 cytokine [80]. These apparently contradicting reports suggest that curcumin may be involved in perturbing complex signaling networks, making its function context-dependant. Curcumin modulates the complex array of signals during the interaction between tumor cells and the immune system to finally leading to an enhanced anti-tumor immunity.

### Reduction of T-regulatory cell population

Another important player in the tumor immune evasion process is the CD4^+^CD25^+^FOXP3^+^ T-regulatory cells (Tregs). These cells in general have an immunosuppressive function and are necessary for prevention of autoimmune disorders [81]. Progression of tumor is associated with an increase in Treg cell population which secrete immunosuppressive cytokines like TGFβ and IL10. Treg not only secrete immunosuppressive cytokines, they also express the high-affinity IL2 receptor CD25, which sequesters IL2 from the tumor milieu. Since IL2 is essential for survival and proliferation of other T cells, unavailability of the cytokine leads to effector T cell apoptosis [82]. The presence of Treg cells in the tumor microenvironment correlates with poor prognosis of cancer [83]. Bhattacharya et al. showed that curcumin can effectively reduce Treg cell population and levels of IL10 and TGFβ [84]. Other studies also reported similar results, showing that pretreatment of CD4^+^CD25^+^ Treg cells with curcumin reduced their immunosuppressive activity [85, 86]. FOXP3 and CTLA4 are two of the key transcription factors that are involved in regulating the Treg transcriptional program and are essential for Treg development and function [87]. This study also showed that curcumin can reduce expression of CTLA4 and FOXP3 both at protein and mRNA levels. Hence curcumin has been shown to modulate the interaction between immune system and tumor cells, restoring the ability of the immune system to successfully eliminate tumor cells.

### Reduced T cell apoptosis

**Fig. 3:**
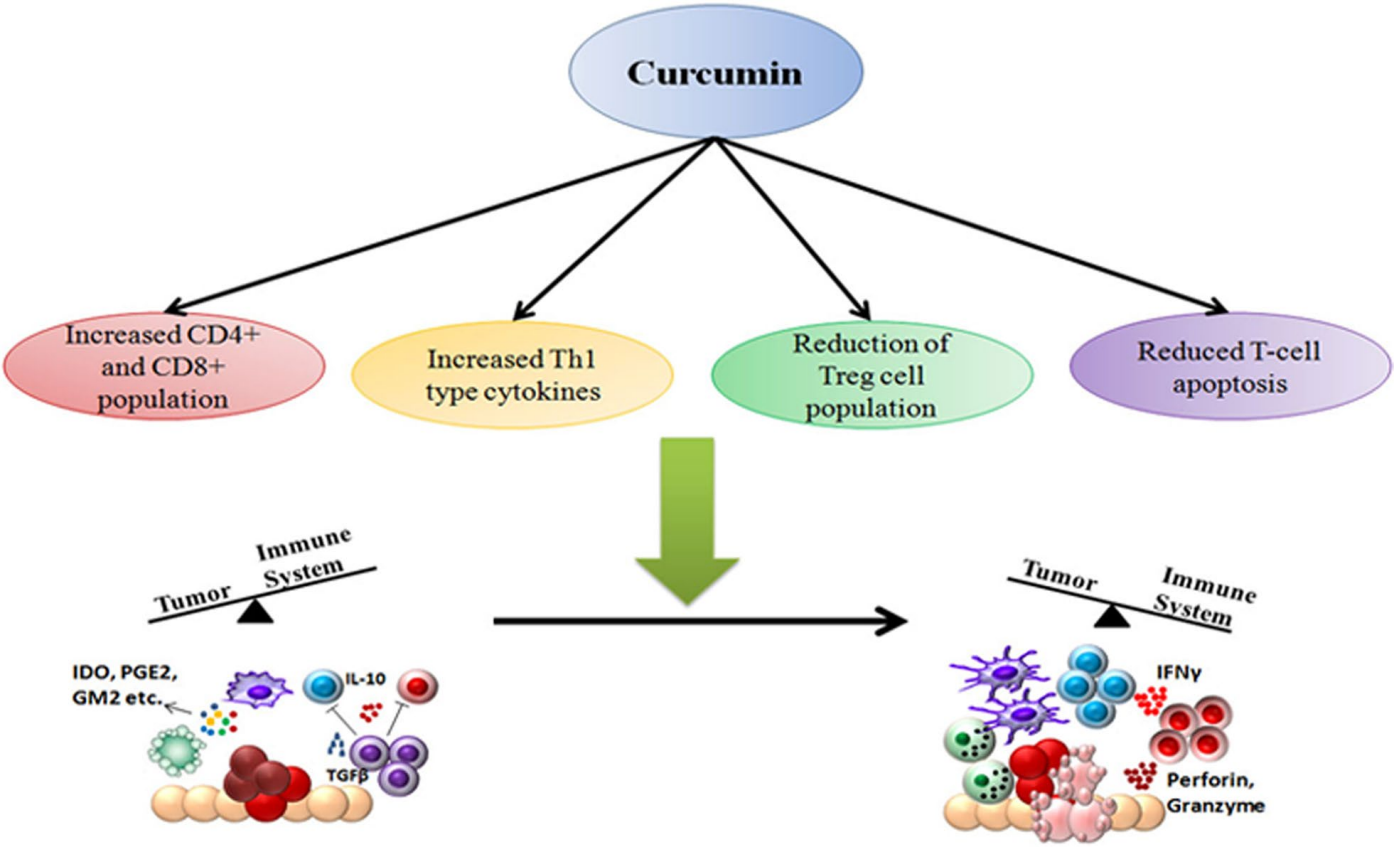
Curcumin enhances anti-tumor immunity: Curcumin can boost anti-tumor immunity through different mechanisms. These include: increased population of CD8^+^, andCD4^+^ T cells, along with increase in Th1 cytokines like IFNγ, which mediate tumor cell apoptosis. Curcumin can block Treg cell development, thereby decreasing immunosuppressive cytokines like IL10 and TGFβ. Curcumin also reduces tumor-induced T cell apoptosis. All these processes help to nullify the overall immunosppressive environment created by tumor and lead to tumor regression. Thus curcumin has the ability to shift the balance in favor of the immune system and reinstate immune system-mediated elimination of tumors

Several other studies also confirmed that curcumin has a positive effect on anti-tumor immunity. Varalakshmi et al. reported that prolonged injections of curcumin did not have any detrimental effects on the immune system; rather they maintained the levels of Th1 cytokine production, NK cell cytotoxic activity and generation of reactive oxygen species and nitric oxide by macrophages [85]. In-vivo studies involving mice bearing ascites carcinoma cells also show similar effects of curcumin on the immune system. It has been shown that administration of curcumin in tumor-bearing mice leads to inhibition of tumor-induced apoptosis in both thymocytes and splenocytes, thereby restoring immune cell numbers and successful regression of tumor [77]. Other studies tried to delineate the molecular mechanisms affected by curcumin in immune cells. The JAK3-STAT5a pathway is responsible for maintaining levels of the anti-apoptotic protein BCL-2 in T cells and its impairment during cancer leads to decreased BCL-2 levels. This in turn increases pro-apoptotic protein BAX, which is responsible for tumor-induced T cell death. It has been reported that curcumin can successfully restore the phosphorylation and activation of the JAK3-STAT5a pathway in T cells and activation of this pathway restores the level of BCL-2, thus reducing T cell apoptosis in tumor-bearing mice [88]. Studies also suggested that curcumin prevents tumor-induced thymic atrophy by restoring activity of the NFκB pathway [89]. Luo et al. reported that the effect of curcumin was dependent on the dose of curcumin administered. Both in vivo and in vitro studies confirmed that a low-dose of curcumin induced effective anti-tumor response by increasing CD8^+^ cytotoxic T cells and IFNγ secretion; whereas a higher-dose of curcumin was detrimental for T cells [90] (Fig. 3).

### Major drawbacks of curcumin

Although curcumin has been used as a most reliable, safe and promising agent with high-efficacy for cancer therapy and chemoprevention but it is not well accepted as a “panacea for all ills” in cancer community. It is feebly soluble in water and it has been reported that solubility of curcumin persisted only approximately 11 ng/ml in aqueous solution (pH = 5.0) [91]. Such poor aqueous solubility creates difficulties in oral administration of curcumin. Curcumin is rapidly hydrolyzed and degraded in neutral and alkaline condition but shows greater solubility in acidic environments. Moreover, rapid metabolism and fast systemic elimination are essential key factors that lead to reduced systemic bioavailability [92–95]. It has been shown that after intraperitoneal or intravenous administration of curcumin, excess amounts of the drug was excreted through bile in the form of tetrahydrocurcumin and hexahydrocurcumin glucuronides derivatives [96, 97]. The reduced bioavailability of orally administrated curcumin in GI tract (i.e. colorectum) limits its therapeutic efficacy against cancer immunosuppression [98, 99]. In a Phase-I clinical trial, colorectal cancer patients at advance metastasis stages were administered 3600 mg of oral curcumin daily, and levels of curcumin and its metabolites were measured by HPLC in portal and peripheral blood [100]. It was found that curcumin was poorly accessible after oral administration, with little amounts (nanomolar levels) existing as the parent compound and its metabolite derivatives like glucuronide and sulphate conjugates in the peripheral or portal circulation. Similarly, in another Phase-I study, 8000 mg of free curcumin were introduced into cancer patients orally per day but only minute levels were detected in portal vein and peripheral systems further highlighting its limitations [101]. In other clinical trial it has been shown that increment of curcumin doses gradually from 500 to 8000 mg/day was not detectable in their bloodstream and only trace amounts of its derivatives were found in the patients who consumed 10,000 mg to 12,000 mg/day [102, 103]. Therefore it is necessary to develop an alternative and efficient strategy to improve solubility and bioavailability of curcumin for a better therapeutic substitute against tumor induced immunosuppression.

## Curcumin nano formulation: future perspectives

Although curcumin acts as a potent immune-modulator, but poor water solubility, low bioavailability, lack of dose–response proportionality, uncontrolled precipitation, use of excessive co-solvents, necessity of extreme condition to solubilize (basic or acidic) and incompatibility to the patients are some of the major hurdles that hampers its efficacy as a chemotherapeutic drug against cancer [104, 105]. To overcome such inconveniences nanotechnology-based drug delivery systems have proven to be most reliable and promising approach. Nanotechnology-based drug delivery systems improve poor bioavailability, enhance biological activities and also selectively target cancer cells. To enhance systematic bioavailability of higher molecular weight drugs, it is now possible to deliver the active pharmaceutical ingredient as reduced nano-sized particles, ranging in size from 10 to 1000 nm. The nanotechnology-based drug delivery system has been proven as a most effective method to successfully deliver insoluble drugs with enhanced bioavailability [106]. The reduction of particle size of active ingredients significantly enhances the dissolution rate resulting in higher bioavailability. Several forms of nanoparticles are being developed for successful encapsulation of curcumin. These include liposomes, nanoparticles, micelles, nanogels, nanoemulsions, nanocrystal suspensions, phytosome complexes, inclusion complexes and dendrimer/dimers [107]. Recently, instead of carrier-based nano formulations, pure curcumin nanoparticles have been developed that are 50 times more effective than normal curcumin, with increased bioavailability. These curcumin nanoparticles restrict tumor-induced Treg cells by inhibiting several Treg markers and restore immune surveillance in tumor-bearing mice [86].

**Fig. 4:**
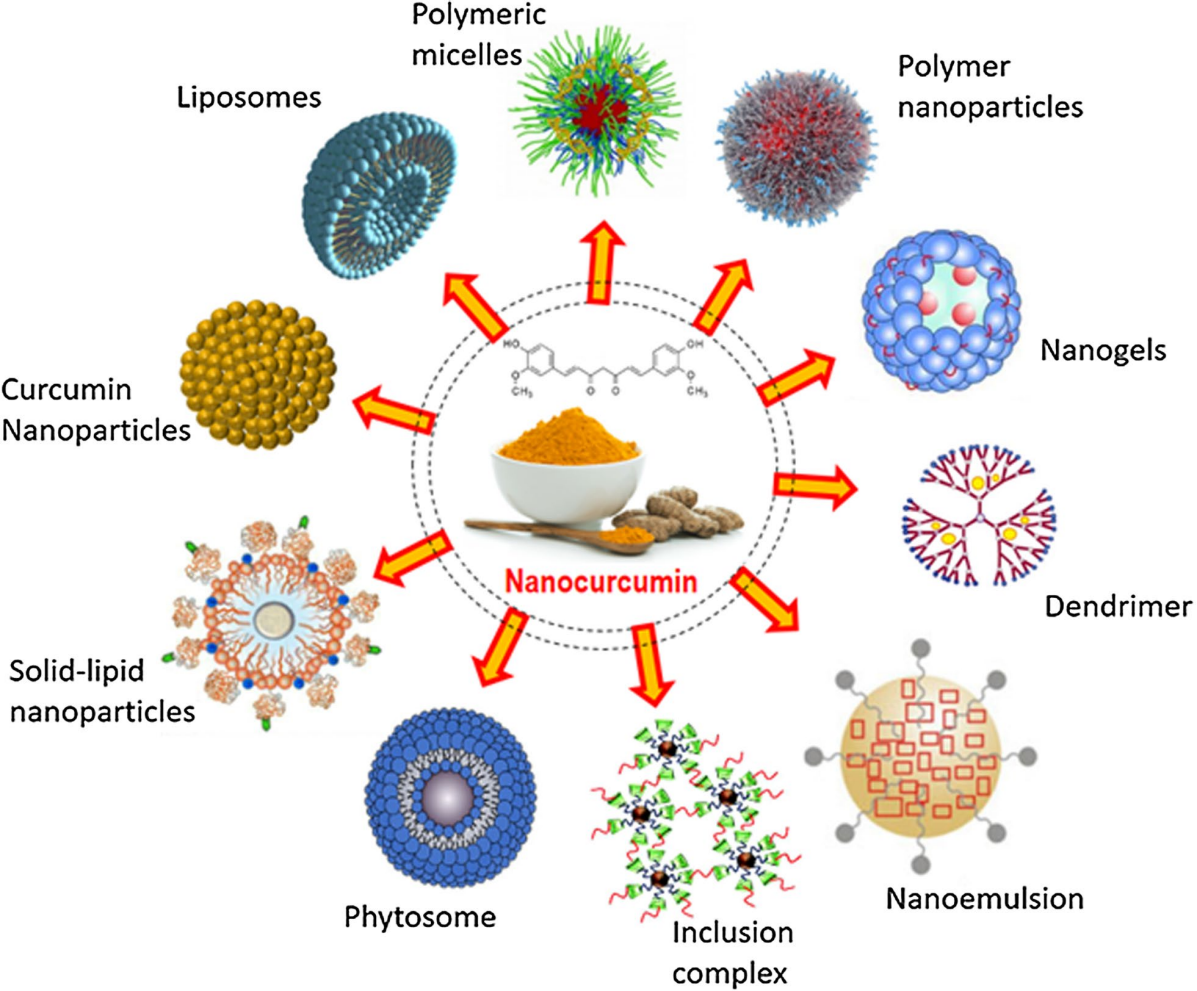
Different strategies of curcumin nano formulation preparation: (1) *Liposomes* Lipophilic particles are incorporated into the hydrocarbon bilayer whereas hydrophilic molecules are incorporated into their aqueous interiors. (2) *Polymeric micelles* They contain both hydrophilic and hydrophobic functional groups and are hence called amphiphiles. They are formed when the concentration of amphiphiles exceeds critical micelle concentration. (3) *Polymer nanoparticles* Consist of intense matrix structure that can incorporate the pharmacologically active ingredients and has a high-drug loading capacity. (4) *Nanogels* A core shell polystyrene gel layer structure consisted of inner hydrophobic core that interacted with active pharmacological substances for high-drug yields and PEG analogue outer shell that trigger fast release of preloaded drug. (5) *Nanoemulsion* Thermodynamically stable dispersion of water and oil, stabilized with active surface film consist of surfactant and cotransfactent. (6) *Solid lipid nanoparticles* consist of solid lipid core matrix that stabilized by surfactants or emulsifier and solubilize lipophilic substances. (7) *Inclusion complex:* mixture of active drug ingredients primarily located in the hydrophobic cavity of bulky host molecules such as cyclodextrin. (8) *Dendrimer* Core–shell nanostructure generally synthesized in layer-by layer fashion where many pharmaceutical active compounds directly associated with stable physical interaction or chemical bonding. (9) Phytosomes: The phospholipid complex, obtained by pure phospholipids containing biological derivatives with active pure ingredients with definite physicochemical and spectroscopic properties. (10) *Curcumin nanoparticles* These are nanoparticles made from pure curcumin without any carrier conjugates. They are prepared by dissolving pure curcumin in ethanol and homogenization at high pressure with water containing 0.1 % citric acid [86]

Although, nanotechnology based drug delivery system has been proven as a major effective and promising approach towards successful cancer therapy but there are also certain limitations. Difficulties such as possibility of drug targeting, drug-loading capacity, in vivo fate of the carrier-molecule conjugates (interactions with the biological microenvironment, rate of disintegration and accumulation in organs), toxic effects of the carrier molecule or its metabolites, its large scale production, stability during long-term storage and overall production costs are difficult to deal with. Especially, the toxic effects of the nano-formulations in the body are a critical parameter. Although the carrier materials are tested for toxicity and biocompatibility, however the properties of the nano particles often differ from bulk material. Hence rigorous and specialised tests for determining the toxicities of the carrier molecules, its metabolites and surfactants are necessary before approval for use [104] (Fig. 4).

## Conclusion

Curcumin is thus found to be an extremely promising anti-cancer agent, targeting various pathways associated with cancer progression. Studies continue to reveal new sides of its mode of action and its interaction with the immune system is emerging as an important contributor to its anti-cancer properties. The need for tumor cells to avoid the immune system during successful tumor progression in the body is now considered to be a new hallmark of cancer. Various studies in the past decade have gradually established curcumin as a potent immune-modulator. Although some reports have suggested a general immunosuppressive role of curcumin and its ability to reduce cell proliferation in immune cell in isolation; specific reports suggest that curcumin boosts anti-tumor immunity through various mechanisms, as discussed in this review. Thus modulation of the immune system seems to be another important strategy by which curcumin counteracts cancer development. This further asserts its effectiveness as an anti-cancer agent and points out the need to develop it as an adjuvant chemotherapeutic agent. This necessitates the development of nano-based strategies for proper delivery and increased bioavailability of curcumin, which may finally lead to its use as a proper chemotherapeutic agent.

## Abbreviations

BCL2: B-cell lymphoma 2
CDK: cyclin dependent kinase
DMSO: dimethyl sulfoxide
EGFR: epidermal growth factor receptor
FLIP: FLICE inhibitory protein
FOXP3: Forkhead Box P3
IκB: inhibitor of κB
IKK: inhibitor of κB kinase
iNOS: inducible nitric oxide synthase
JAK: Janus kinase
JNK: cJUN N-terminal kinase
MAPK: mitogen-activated protein kinase
mTOR: mammalian target of rapamycin
NF-κB: nuclear factor κB
PI3K: phosphatidylinositol-3-kinase
PKC: protein kinase C
PRB: retinoblastoma protein
PUMA: P53 upregulated modulator of apoptosis
STAT: signal transducer and activator of transcription
Th1: T-helper1
Th2: T-helper 2
Treg: T regulatory cells
VEGF: vascular endothelial growth factor
XIAP: X-linked inhibitor of apoptosis
